# Intrinsic PPG–ECG Coupling for Accurate and Low‐Power Blood Pressure Monitoring

**DOI:** 10.1002/advs.202520101

**Published:** 2026-03-03

**Authors:** Sitong Chen, Hua Luo, Zhentao Yao, Zhou Jiang, Xiaodong Wu, Hanmin Liu

**Affiliations:** ^1^ Department of Pediatric Pulmonology and Immunology West China Second University Hospital Sichuan University Chengdu China; ^2^ School of Mechanical Engineering Sichuan University Chengdu China; ^3^ NHC Key Laboratory of Chronobiology Sichuan University Chengdu China; ^4^ Key Laboratory of Birth Defects and Related Diseases of Women and Children Sichuan University Ministry of Education Chengdu China; ^5^ Department of Pediatric Pulmonology and Immunology WCSUH‐Tianfu·Sichuan Provincial Children's Hospital Sichuan University Meishan China; ^6^ The Joint Laboratory for Lung Development and Related Diseases of West China Second University Hospital Sichuan University and School of Life Sciences of Fudan University West China Institute of Women and Children's Health West China Second University Hospital Sichuan University Chengdu China; ^7^ Sichuan Birth Defects Clinical Research Center West China Second University Hospital Sichuan University Chengdu China

**Keywords:** blood pressure, electrocardiogram, photoplethysmography, pulse transit time, wearable sensor

## Abstract

Long‐term, continuous blood pressure (BP) monitoring is of great clinical importance for the early diagnosis and dynamic assessment of cardiovascular diseases. Conventional BP monitoring methods based on pulse transit time (PTT) typically rely on at least two independent signal channels. However, multi‐signal processing introduces synchronization errors and inaccuracies in fiducial point detection, thereby limiting the accuracy and efficiency of BP estimation. In addition, recording and processing multiple signals increases circuit complexity and elevates system power consumption. To overcome these limitations, we propose an intrinsic signal synchronization strategy based on photoplethysmography–electrocardiogram (PPG–ECG) signal coupling. By fusing PPG and ECG signals into a unified waveform that preserves the essential features of both, intrinsic synchronization is inherently achieved. Compared with conventional independent signal processing, this approach reduces PTT error to less than 15 ms and achieves a PTT detection rate exceeding 98%. Furthermore, the data load is significantly reduced, resulting in a 43.75% decrease in system power consumption. Based on this principle, we developed a compact, lightweight, chest wearable sensor patch. The device demonstrates high BP measurement accuracy (ME ≤ 5 mmHg, STD ≤ 8 mmHg) across diverse scenarios, including both static and dynamic BP conditions. This strategy not only simplifies the design and manufacturing of BP monitoring devices but also enhances their performance in terms of accuracy, energy efficiency, and operational lifetime.

## Graphical abstract

A PPG–ECG coupling strategy for continuous blood pressure monitoring that intrinsically synchronizes signals within a single waveform is demonstrated, minimizing synchronization errors and hardware complexity. This approach halves power consumption while maintaining high accuracy, enabling compact, energy‐efficient wearable devices for personalized cardiovascular health management.

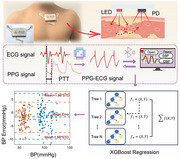

## 1 Introduction

Blood pressure (BP) serves as a crucial indicator for cardiovascular health, playing a pivotal role in the detection and treatment of cardiovascular diseases [1, 2]. In clinical practice, BP is typically measured with conventional cuff‐based sphygmomanometers, which only provide intermittent, static readings. This traditional method does not support continuous monitoring during daily activities or ambulatory settings, limiting its utility for continuous and remote patient management. Continuous BP monitoring is increasingly recognized as essential for the timely diagnosis and treatment of diseases, aligning with the precision‐oriented requirements of modern clinical care [3, 4, 5].

In recent years, wearable electronic devices for continuous BP monitoring have gained significant momentum, aiming at home‐based health monitoring and accurate vital sign assessment [6, 7, 8, 9]. Among these devices, cuffless wearable BP monitoring has emerged as a main research topic, attracting extensive investigations [10, 11, 12, 13, 14, 15, 16, 17]. Various cuffless techniques have been explored, including ultrasound wall‐tracking [18, 19], piezoelectric sensing [20, 21], impedance plethysmography (IPG) [22, 23], and photoplethysmography (PPG) [24, 25, 26, 27, 28]. The ultrasound‐based method utilizes a high‐precision probe to capture the cross sectional image of blood flow and estimate BP based on hemodynamic parameters. However, this method involves complex signal propagation and processing. In addition, the portable ultrasound probe—due to its size constraints—typically has relatively weak signal intensity, imposing high requirements on the stability of signal acquisition [29]. The piezoelectric method uses piezoelectric sensors closely adhered to the skin to detect changes in arterial pulse pressure, converting biomechanical signals into electrical signals and generating a measurable pulse waveform. However, accurate measurement of the pulse waveform is challenging owing to its subtlety, which is highly susceptible to wearing pressure and skin vibration. This can bring artifact signals aliasing in the pulse waveform [30, 31]. IPG signals, which reflect changes in vascular volume, are particularly vulnerable to interference from tissue conductivity variations and electrode–skin contact conditions. These factors can cause signal degradation and calibration drift, resulting in limited clinical acceptance of impedance‐based BP monitoring due to its limited reliability [32].

PPG has gained significant attention due to its compact design and suitability for wearable devices. Optical BP measurement based on PPG involves calculating the pulse transit time (PTT) by independently processing PPG and electrocardiogram (ECG) signals or multiple PPG signals. However, the PPG waveform can be highly affected by individual differences (e.g., skin color) and environmental noises, which necessitate robust algorithms for accurate detection of fiducial points in PPG waveforms [33, 34, 35, 36, 37, 38, 39]. The R waves with a high signal‐to‐noise ratio (SNR) in ECG signals can provide a stable starting point for PTT calculation. BP estimation in wearable monitoring mainly relies on the correlation of PPG and ECG signals. These approaches can be grouped into two categories. The first involves data integration of PPG and ECG, where the two separately acquired signals are integrated into a multidimensional data matrix that supplies multiple features for the BP model [40, 41, 42]. Data integration can be performed directly on raw signals [43] or after a frequency‐domain transformation [44]. However, this method merely integrates the two signals at the data level after separate signal processing and transmission, rather than achieving natural signal fusion at acquisition. As a result, strict temporal synchronization between PPG and ECG signals cannot be guaranteed. The other category is signal inferring [45, 46], which trains models using large amounts of synchronous PPG and ECG data to learn their correlations. The trained model can infer the primary features of both signals from a single signal. This method includes reconstructing one physiological signal from another [47], as well as learning the features of two synchronized signals from a single signal [48]. Nonetheless, this approach relies heavily on the strong correlation learned from the training data. During application, any deviation from training conditions (e.g., individual variability) may cause inaccurate inference and reduce the reliability of BP estimation.

To resolve these limitations, we introduced a new intrinsic PPG‐ECG coupling method to achieve natural signal fusion at the source. This coupling method combines PPG and ECG signals into a single coupled waveform that retains both PPG and ECG characteristics, which brings about three significant advantages compared to conventional BP monitoring methods. First, the PPG‐ECG coupling can enhance fiducial point detection reliability in PPG signals and enable accurate calculation of vital cardiovascular parameters such as PTT. Second, coupling PPG and ECG signals ensures precise temporal alignment and avoids timing errors often arising when processing two signals separately. Thirdly, since only one signal is acquired, transmitted and processed, the complexity and power consumption of the system are significantly reduced, which is significant for wearable electronic devices.

We also developed a sensor patch system for continuous BP monitoring based on this new principle and methodology. This wearable sensor patch enables synchronous acquisition of ECG and PPG signals, eliminating multi‐sensor complexing. The distinctive waveform features of the PPG‐ECG coupled signal can be processed with a multi‐feature machine learning model to achieve accurate BP prediction. We show that the PPG‐ECG coupling method enables us to detect and calculate PTT with high precision. The mean absolute error of PTT is under 15 ms, and transmission power consumption is reduced by 43.75%. The BP monitoring system enhances BP estimation stability and accuracy through a new signal coupling method, achieving high accuracy in tests and meeting AAMI standards (mean error ≤ 5 mmHg, standard deviation ≤ 8 mmHg), proving reliable for home use. Compared with other BP monitoring devices (Table S1), our device exhibits obvious superiority in terms of system integration level, signal processing methods, and BP estimation accuracy.

## 2 Results and Discussion

### 2.1 General Design Principle

**FIGURE 1:**
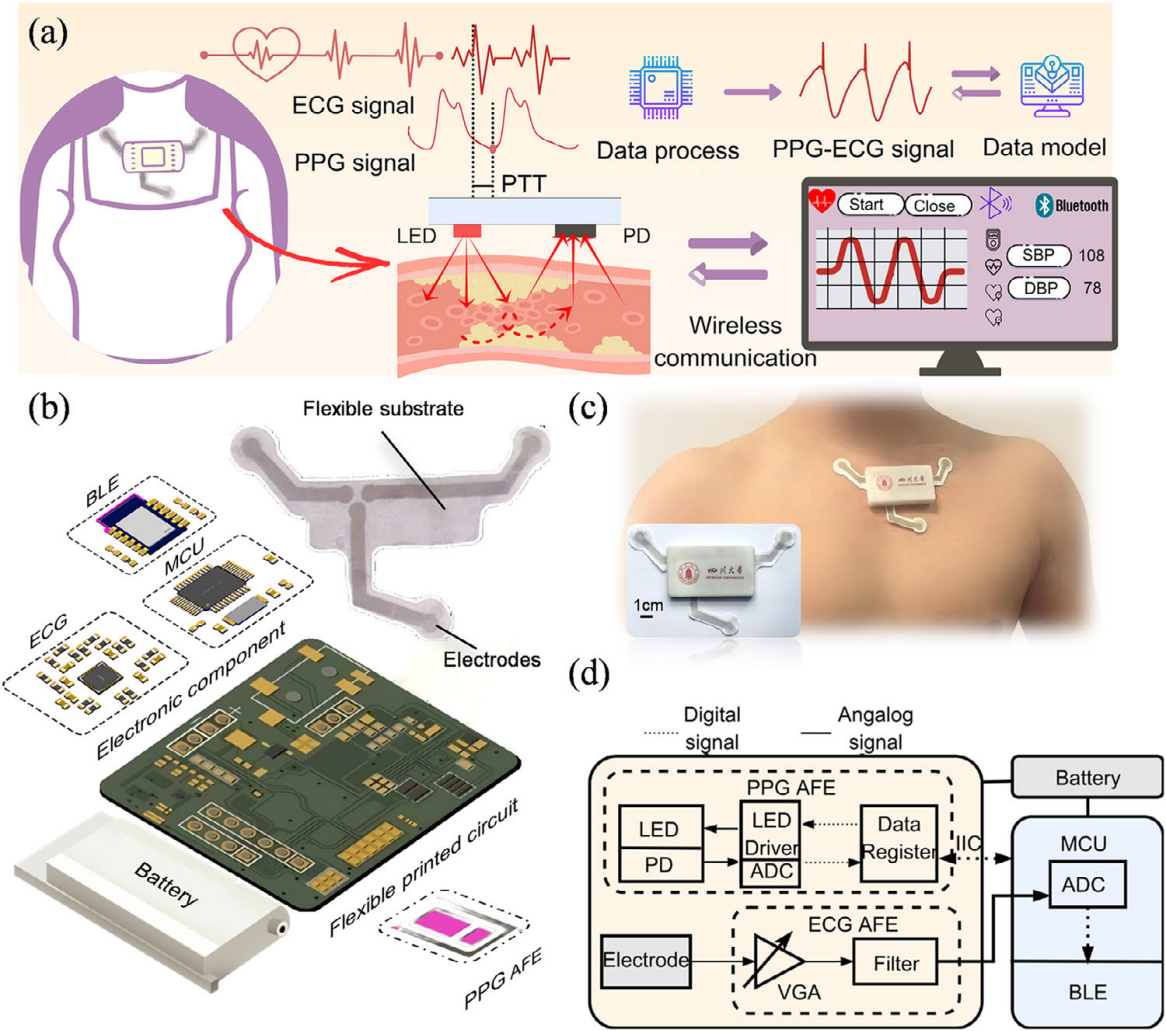
Overall design and working principle. (a) Schematic diagram of a chest wearable sensor patch for BP monitoring, which synchronously acquires ECG and PPG signals, followed by subsequent signal processing, coupling, and wireless transmission to a terminal for continuous BP estimation. (b) Explosive view of the chest wearable sensor patch. The sensor patch is vertically stacked from the bottom (in contact with the skin) to the top in the following sequence: PPG sensor, ECG electrodes on a fabric substrate (skin‐contact interface), and a patch with an fPCB circuit and power supply. (c) Optical images of the sensor patch. (d) Block diagram illustrating the signal flow of PPG and ECG signals in the circuit of the sensor patch. The original signals are acquired and processed by the AFEs, coupled by an MCU, and wirelessly transmitted via BLE to a terminal device for further analysis and display.

Conventional wearable BP monitoring systems based on PTT record and process two independent PPG and ECG signals. The R wave of the ECG signal serves as the starting point, and a specific fiducial point (usually the systolic peak or onset) of the PPG signal is used as the end point [49, 50, 51, 52, 53]. However, this routine leads to high circuitry complexity, high power consumption, and low BP prediction accuracy. To overcome these issues, we propose a PPG‐ECG coupling strategy by superposing and hybridizing PPG and ECG signals into a single signal with inherent features from the two original signals. Using the coupled single signal, BP‐related characteristics such as PTT can be continuously and precisely calculated, and ambulatory BP can be predicted via a machine learning model (Figure 1a).

Based on the PPG‐ECG coupling strategy, we design a flexible, ultralight, and compact chest wearable sensor patch. The patch comprises a photoelectric component, three ECG electrodes, a battery, and basic electronic components on a flexible printed circuit board (fPCB) (Figure 1b). To enhance robustness, the fPCB and battery are encapsulated within a 3D‐printed enclosure for protection (Figure 1c), which is then mounted on a fabric substrate to improve user comfort. The entire patch weighs only ≈15 g (Figure S1). The photoelectric component includes LEDs and photodiodes (PDs), along with an integrated analog front end (AFE) to obtain the PPG signal from the skin of the chest. Meanwhile, the three electrodes attached to the chest can capture high‐quality ECG signals with high‐fidelity R waves. The acquired PPG and ECG signals are then coupled by the microcontroller unit (MCU) (Note S1 and Figure S2). This method achieves the natural synchronization of the two signals through a coupling and processing sequence. It reduces the timing errors caused by sensor transmission, thereby improving the accuracy of the BP estimation.

Figure 1d shows the signal processing and transmission pathway. Synchronously collected PPG and ECG signals from the chest are processed by AFE and combined by the MCU with the signal coupling method, which superimposes their characteristic in real time to create the coupled PPG‐ECG signal (Notes S2 and S3). This signal is then transmitted via BLE to a terminal device (e.g., a personal computer or smartphone) for the calculation of high‐precision BP‐related parameters and finally for estimating real‐time BP.

### 2.2 Principle of ECG‐PPG Signal Coupling

**FIGURE 2:**
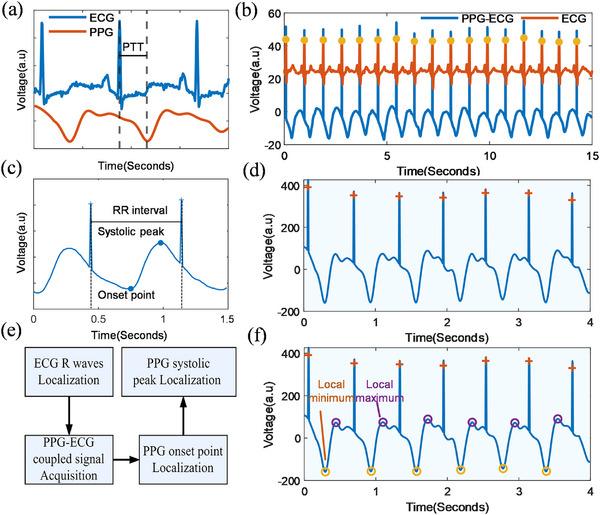
Principles of signal coupling and PPG fiducial points detection. (a) Determination of the PTT, the time difference between the PPG and ECG signals. All signals were acquired with the volunteer in a seated, resting position unless otherwise stated. The ECG was recorded in a simulated Lead I configuration with electrodes on the chest, and the PPG sensor was located below the right collarbone. (b) Comparison between the PPG‐ECG coupled signal and the original ECG signal, showing that the coupled signal accurately preserves the temporal characteristics of the source signal. (c) Illustration of the coupled PPG‐ECG signal with key fiducial points (including the ECG R wave, PPG systolic peak, and onset point). (d) Identification of high‐SNR R waves via PPG‐ECG signal coupling. (e) Flowchart for extracting vital fiducial points from the coupled PPG‐ECG signal. (f) Identification of PPG fiducial points anchored to the ECG R waves. The PPG‐ECG coupled signal uses R waves of the ECG signal as reference points to locate PPG onset points (local minimum) and systolic peaks (local maximum) within each cardiac cycle.

The definition of PTT is the time required for a pulse to travel between two sites in the artery. As a non‐invasive physiological marker, it reflects cardiovascular status. Fluctuations in BP alter vascular wall stiffness, thereby altering PTT [54]. The R waves in the ECG signals represent the electrical activation of the ventricles and occur before their mechanical systole. In contrast, PPG waveforms result from the mechanical pumping of blood into the aorta during ventricular systole and diastole [55]. Therefore, the fiducial point of the PPG signal lags behind the R wave of the ECG signal within each cardiac cycle. The fiducial points (systolic peak and onset) of the PPG signal and R waves of the ECG signal always appear in pairs per cycle. Calculating PTT usually requires separate ECG and PPG signals (Figure 2a). Here, we introduce a new method to integrally couple ECG and PPG signals without losing their main features. By coupling the PPG and ECG signals via superposition during acquisition, the system transmits and processes only the coupled signal, as detailed in the Methods section. Consequently, it allows us to continuously and easily calculate BP by extracting key fiducial points from the single coupled PPG‐ECG signal, including the R waves of ECG, as well as the onset points and systolic peaks of the PPG.

In addition to the PPG‐ECG signal coupling method, we propose a key feature extraction algorithm from the PPG‐ECG coupled signal. The R waves of the ECG signal were retained to ensure that the phase of the coupled signal is consistent with the original ECG signal, as shown in Figure 2b. The pre‐processing of the original signal and the coupling process are described in the Methods section. The PPG‐ECG coupled signal is temporally aligned with the original signals. The coupled signal contains the features of both ECG and PPG signals, which facilitates calculation of time‐series features such as RR intervals and PTT, and enables natural synchronization of the two signals (Figure 2c). The R waves with a relatively high SNR can be accurately extracted through a dynamic difference method (Figure 2d; and Note S4). Using the R waves as the anchor points, the onset point can be detected using the local minimum method within the RR interval. Subsequently, the systolic peak can be identified by searching for the local maximum between the onset point and the next R wave (Figure 2e,f).

### 2.3 Advantages of ECG‐PPG Signal Coupling

The main advantages of the proposed PPG‐ECG coupling method are as follows. First, the two biological signals are coupled before processing and transmission, which reduces the time delay caused by independent signal processing and transmission. Second, PPG‐ECG coupling enables natural synchronization of the two signals without timing errors. Thirdly, leveraging the relative temporal relationship between the R wave of high SNR and the pulse fiducial points, the accuracy of PTT calculation can be substantially improved. Fourthly, transmitting a single coupled signal rather than two independent PPG and ECG signals reduces BLE transmission power (Figure S3).

Eight volunteers were recruited to validate the advantages of the PPG‐ECG coupled method, and a dedicated dataset was created from their coupled signals (Note S5). On the dataset, the performance of the proposed PPG–ECG coupling method was compared with two established methods based on individual signal: local extrema detection without R waves anchoring [56, 57] and adaptive derivative analysis (Notes S6 and S7) [58, 59]. The accuracy of fiducial point detection and PTT calculation is evaluated separately.

**FIGURE 3:**
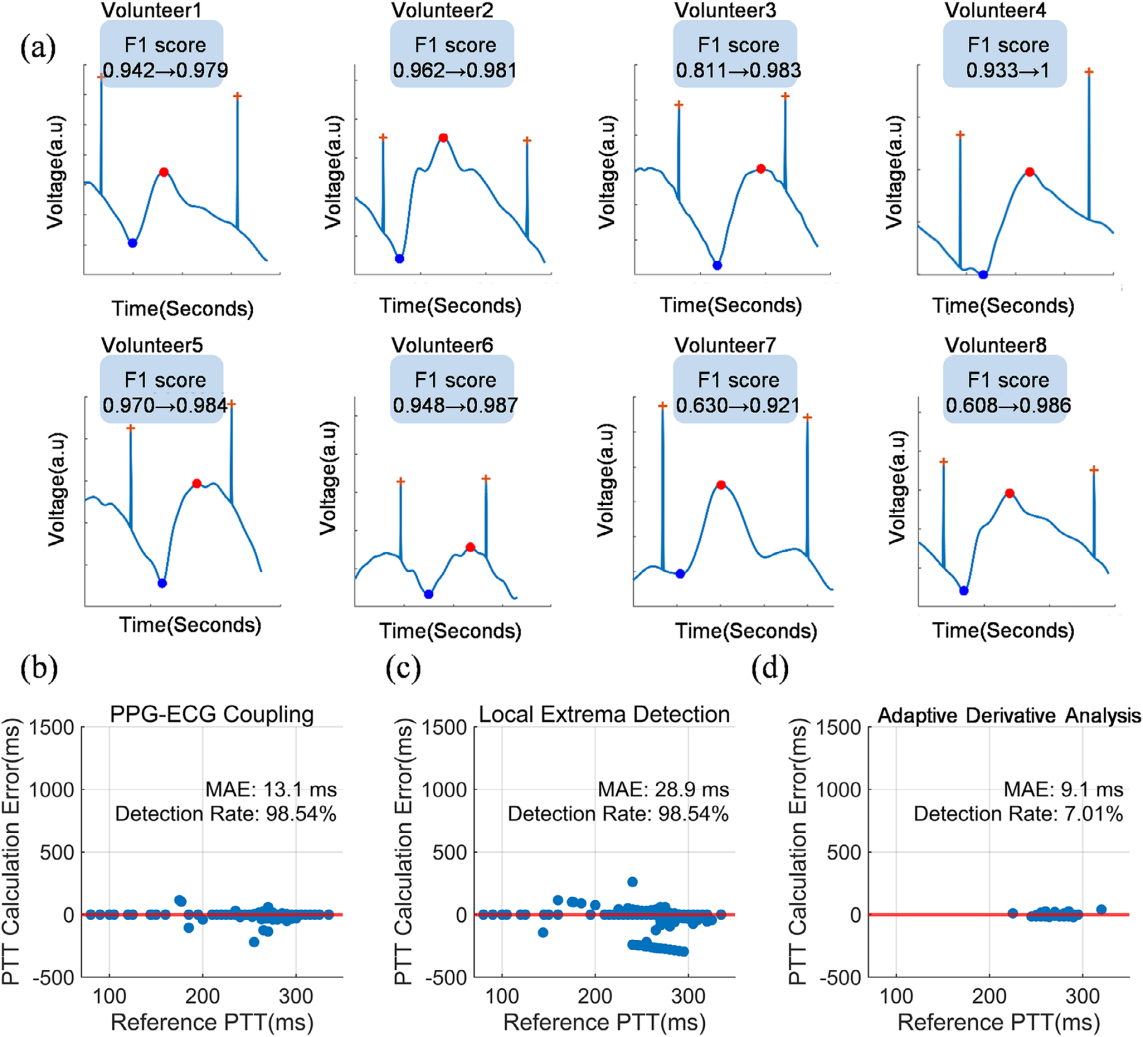
Fiducial points detection and performance comparison. (a) Coupled PPG‐ECG signals acquired from eight volunteers. Each subplot shows F1 score improvement for systolic peak detection. (b–d) Scatter plots of PTT calculation errors against reference PTT values for (b) PPG‐ECG coupled method (*n* = 2560), (c) local extrema detection (*n* = 2560), and (d) adaptive derivative analysis (*n* = 182). The PPG‐ECG coupled method had significantly lower PTT errors (Kruskal‐Wallis test, *p < 0.05; post hoc Mann‐Whitney U test, *p < 0.05).

F1 score is selected as the accuracy evaluation criterion for fiducial point detection, which serves as a comprehensive indicator of precision and recall (Note S8). Figure 3a illustrates the PPG‐ECG coupled signals of 8 volunteers, along with the fiducial point identification of each volunteer during one cardiac cycle. Our proposed method consistently achieves an F1 score > 0.92 across different pulse waveforms. Detection results for fiducial points from the database are summarized in Tables S2 and S3. The individual signal methods achieved average F1 scores of 0.714 for the derivative analysis method and 0.776 for the local extrema detection method. In contrast, the coupling method significantly improved the accuracy, attaining an average F1 score of 0.962. This improvement in fiducial point detection accuracy stems from the anchoring and reference effect provided by the R waves. Such anchoring and reference effects enable this method to adapt to various interfering pulse waveforms and significantly enhance the detection accuracy and robustness of fiducial points.

Moreover, the PTT calculation errors and PTT detection rates for the PPG‐ECG coupling method and the two individual signal methods are compared in Figure 3b–d. The PPG‐ECG coupling method results in the top performers, yielding a mean absolute error (MAE) less than 15 ms and a detection rate of more than 98%. The superiority of the PPG‐ECG coupling method is explained below. Since the calculation of PTT requires pairs of R waves and onset points in the same cardiac cycle, any missing detection of an onset point will lead to incorrect matching of the fiducial points and cause an error in PTT calculation. Furthermore, the individual signal processing methods have poor adaptability to varying pulse amplitude and frequency characteristics, which also leads to higher detection errors and lower detection rates. The PPG‐ECG coupling method can address these challenges via R waves anchoring, which gives rise to higher detection accuracy. We also confirm the advantages of the PPG‐ECG coupling method in the public database (Note S9). The signal coupling method can improve the identification quality of fiducial points and the accuracy of PTT calculation.

### 2.4 BP Prediction Based on ECG‐PPG Signal Coupling

**FIGURE 4:**
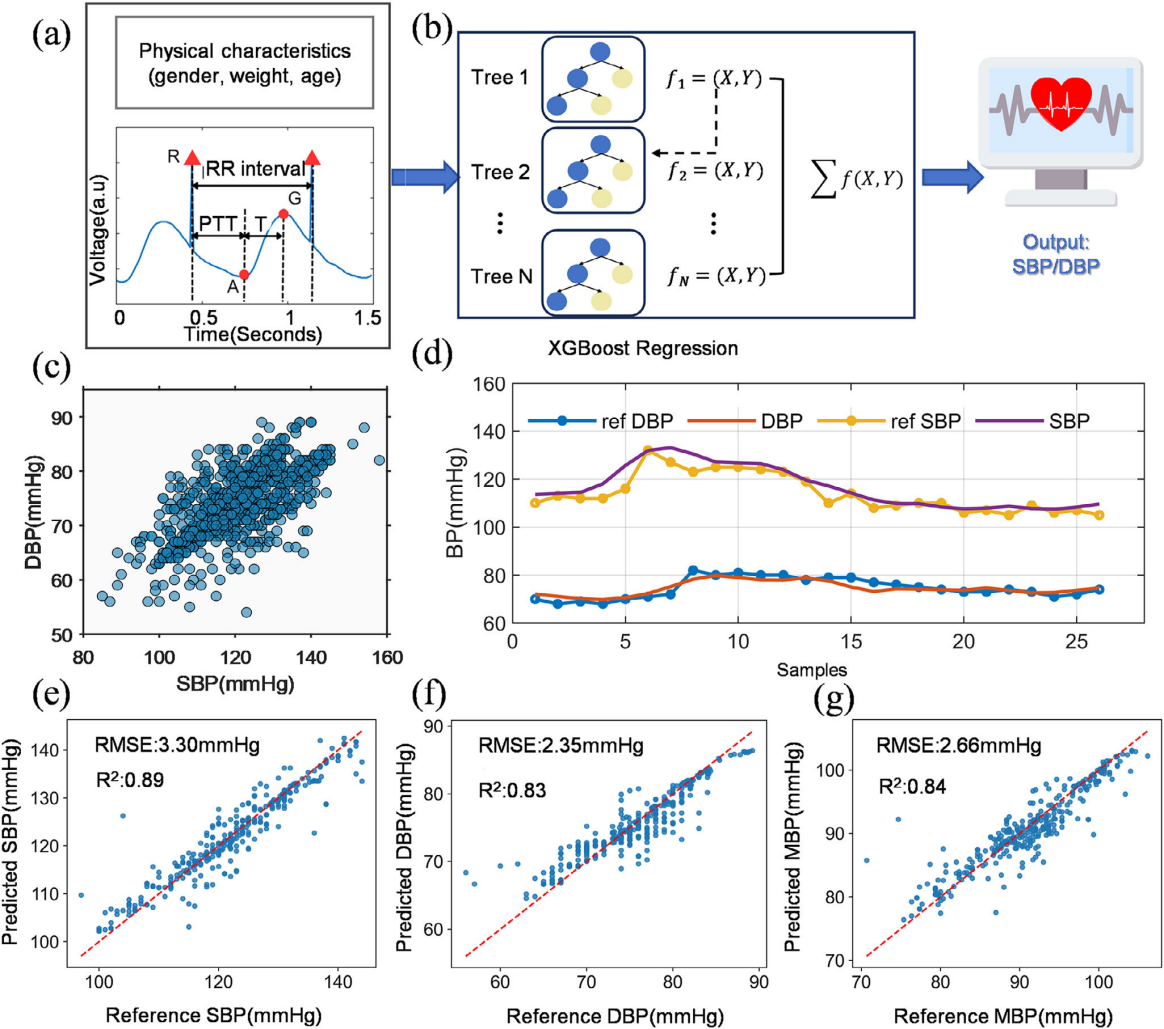
BP prediction via PPG–ECG coupling method. (a) Illustration of extracted features from the coupled PPG‐ECG signal, including the RR interval, PTT, PPG intensity ratio (PIR), and the time interval from the pulse onset point to the systolic peak (denoted as T). (b) BP estimation using an XGBoost model input with the waveform features and physical characteristics. (c) Scatter plot of SBP and DBP from 24 volunteers with a wide dynamic range, enabling robust BP estimation across diverse conditions (*n* = 1574). (d) Evaluation of the BP estimation model with a single volunteer's test set, comparing predicted and reference BP values over a complete measurement cycle. Scatter plots comparing predicted and reference BP estimates for systolic (e), diastolic (f), and mean arterial pressure (g) using test set data from 5 volunteers (*n* = 328). The RMSE and R^2^ were calculated for each model.

Methods for obtaining BP via PTT primarily include physical models and machine learning. Physical models link PTT to arterial wall stiffness via the Moens‐Korteweg equation, and BP is highly correlated with arterial wall stiffness. This method relies on simplified vascular assumptions and requires frequent calibration. Meanwhile, machine learning methods improve the accuracy and robustness of BP estimation by learning the complex relationship between PTT and BP from data [59]. Conventional BP prediction models typically use a single feature (e.g., PTT), which often suffer from poor robustness due to limited feature representation [60, 61, 62]. Here, we proposed a multi‐feature BP estimation model. This model leverages multiple morphological features (e.g., PPG intensity ratio, RR interval, PTT) extracted from the coupled PPG–ECG signal and other physical characteristics (e.g., gender, weight, age) as the inputs to achieve higher robustness and accuracy, as illustrated in Figure 4a. More discussion is presented in Note S10.

Machine learning models capture the complex relationship between multi‐modal features and BP. To avoid overfitting, which is prevalent in large‐scale models, we prioritize a simpler architecture that offers better generalization. Here, we adopt an ensemble Extreme Gradient Boosting (XGBoost) model due to its good balance between simplicity and regression performance (Figure 4b). XGBoost, an advanced form of Gradient Boosted Trees, can aggregate weak learners into a robust final model. XGBoost can also handle diverse data without extensive pre‐processing and provide essential parameter metrics for model interpretability. Hyperparameter optimization was performed using Bayesian optimization with the tree‐structured Parzen estimation. The objective is to maximize the coefficient of determination (R^2^). The search encompassed key parameters including the number of trees, learning rate, maximum tree depth, minimum child weight, L1 and L2 regularization terms, regulation parameter gamma, subsample ratio, and feature sampling ratio (Figure S6). The PPG‐ECG coupled signal can be continuously recorded by the chest wearable sensor patch for dynamic BP monitoring. Meanwhile, the reference BP is measured synchronously using a cuff‐based sphygmomanometer for comparison. 24 volunteers (age: 29.50 ± 9.43, 13 male and 11 female) were recruited to study the BP variations during the continuous BP measurement. Blood flow and BP can be temporarily increased through exercise. The changes in post‐exercise BP relative to the baseline level were also measured. The BP distribution is presented in Figure 4c, with a wide range of BP distribution from 53 mmHg to 158 mmHg (Figure S7). Such a large sample size and wide data distribution are prerequisites for mitigating overfitting during model development. Figure 4d shows the variation in predicted BP (including both systolic BP (SBP) and diastolic BP (DBP)) from our sensor patch, along with the variation in reference BP measured with a commercial sphygmomanometer over a measurement cycle, demonstrating the high reliability and accuracy of our sensor patch.

Figure 4e–g presents the scatterplots of the predicted BP measured by our sensor patch and the reference BP measured by a commercial sphygmomanometer. The coefficients of determination (R^2^) are 0.89 (for SBP), 0.83 (for DBP), and 0.84 (for mean BP, MBP). The RMSE values were 3.30 mmHg (for SBP), 2.35 mmHg (for DBP), and 2.66 mmHg (for mean BP). These results indicate that BP measurement using our proposed signal coupling method and multi‐feature BP prediction model has a high correlation with the practically measured BP with a commercial sphygmomanometer. Such high accuracy and reliability of BP measurement based on a single PPG‐ECG coupled signal is compatible or even superior when compared to other reported wearable BP measurement devices [63, 64, 65, 66, 67]. Notably, reliable BP estimation is maintained even with short‐distance PTT measurement. By measuring the PPG signal at the chest, we capture the pulse wave after its propagation through central arteries. As shown in Figure S12, the pulse travels predominantly through the elastic arterial segment rather than the muscular arterial segment before reaching the subcutaneous vasculature of the chest [68]. Consequently, although the PTT measured at the chest is small in absolute value, it is closely related to the properties of elastic arteries and minimizes the effect of vasomotion. Furthermore, the temporal error between the two signals is reduced through an intrinsic synchronization between the PPG and ECG signals, thus minimizing the error in the calculation of BP‐related features.

### 2.5 Application in Ambulatory BP Monitoring

**FIGURE 5:**
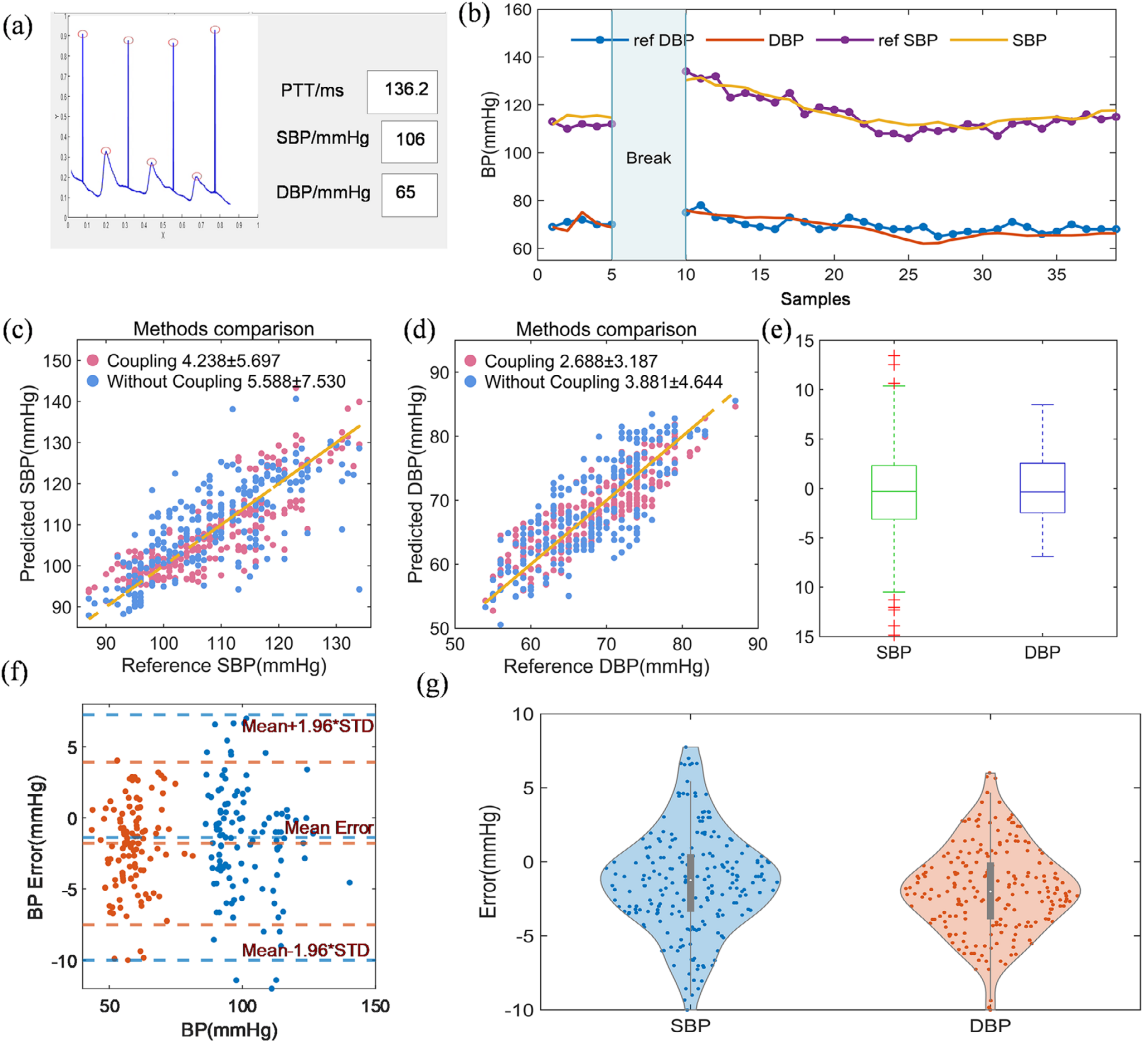
Performance evaluation of the chest wearable sensor patch with PPG–ECG coupled signal. (a) User interface of the BP monitoring system. (b) Continuous BP estimation versus reference values from a volunteer during a single test session, including an exercise interval (“Break”). (c,d) Scatter plots of estimated versus reference values for SBP and DBP, respectively, comparing the PPG‐ECG coupling method (blue) and individual signal processing (pink) (*n* = 221 per method). The coupling method shows significantly lower error (Wilcoxon signed‐rank test, *p < 0.05 for both SBP and DBP). (e) Box plot of ME for the coupling method. (f) Bland‐Altman and (g) violin plots representing the ME of BP measurements over a 5‐day evaluation period (*n* = 192).

To verify the effectiveness and generalization ability of our sensor patch with PPG–ECG coupled signal for ambulatory BP measurement, 10 healthy volunteers were recruited. We collected 221 BP measurement datasets from the 10 volunteers for dynamic BP evaluation. The real‐time BP values measured by the sensor patch are detected and displayed (Figure 5a), meanwhile, a commercial sphygmomanometer is used to measure the reference BP, as illustrated in Figure S8 and Movie S1. Each measurement cycle consists of three phases: 5 samples of resting BP recording, 5 min of exercise‐induced BP elevation, and 15–20 samples of BP recovery to baseline levels. Details are provided in the Methods section. Figure 5b compares the BP results continuously recorded using the sensor patch and discrete data points detected via a commercial sphygmomanometer, which exhibits high consistency and confirms the high reliability of our sensor patch with PPG–ECG coupled signal.

Based on the above dataset, we evaluated the performance of the BP prediction models using two feature sets: one set of features derived from the signal coupling method and the other from individual signal processing. We also trained an XGBoost model with features extracted from individual signals via the local extrema method. Estimated BP values were statistically assessed using the MAE and standard deviation (STD), as defined in Note S11. Figure 5c,d summarizes the results of BP prediction with PPG–ECG coupled signal combined with incorporated physical parameters in the XGBoost model, showing a prediction error of 4.238 ± 5.697 mmHg for SBP and 2.688 ± 3.187 mmHg for DBP. Figure 5e illustrates the distribution of BP estimation errors for 221 samples collected via our sensor patch with PPG–ECG coupled signal. The median errors for SBP and DBP are ‐0.285 mmHg and 0.390 mmHg, with distributed positive and negative errors. The individual signal processing method provided a median error of 1.544 mmHg for SBP and 1.047 mmHg for DBP (Figure S9). These results indicate that the PPG–ECG coupling method can effectively improve the accuracy of BP estimation. This can be attributed to the fact that the PPG–ECG signal coupling method can eliminate the time synchronization error and reduce the feature extraction error.

To assess the long‐term stability of the chest wearable sensor patch, the volunteers were invited to measure their BP using our device and an electronic sphygmomanometer. The measurements were conducted in the morning and evening, respectively, which lasted for 5 consecutive days. Figure 5f shows the Bland‐Altman plot of the BP estimation errors in the long‐term study. The proportions of SBP and DBP samples within the limits of agreement (LoA; mean error ± 1.96 × STD) were 96.0% and 96.5%, respectively. This result indicates that the BP estimated by our device in long‐term measurements agrees with the practically measured BP with a commercial sphygmomanometer. Figure 5g presents the violin plot of the error distribution. The mean error (ME) values were −1.7977 and −1.3677 mmHg for DBP and SBP, respectively. The STD values were 4.406 and 2.917 mmHg for DBP and SBP, respectively. Such low BP estimation errors for the SBP and DBP met the AAMI standard (ME ± 5 mmHg and STD ± 8 mmHg) [69].

## 3 Conclusions

In summary, we present a PPG–ECG coupled method to detect key features related to PTT for continuous BP monitoring. The method couples PPG and ECG signals to eliminate temporal misalignment caused by processing and transmission delays between different sensors, thereby achieving natural synchronization of the two signals. The anchoring effect of the R waves provides a stable temporal reference, which improves the accuracy and robustness of the detection of fiducial points. This enhancement leads to more accurate and reliable calculation of the hemodynamic parameters (e.g., PTT), thereby enhancing the accuracy of BP estimation. Furthermore, a chest wearable sensor patch was developed based on the PPG‐ECG coupled method. With this device, we established a multi‐parameter BP estimation model and validated the performance of BP estimation through comparative experiments. The results demonstrate that the proposed approach improves the accuracy and detection rate of PTT calculation and enhances the accuracy of BP measurements while reducing overall power consumption by 43.75%. Therefore, the proposed intrinsic PPG‐ECG coupling method and the developed chest wearable sensor patch enable long‐term and real‐time BP monitoring, demonstrating potential for ambulatory BP monitoring.

## 4 Methods

### 4.1 Flexible Printed Circuit Board and Wireless Transmission

The sensor patch circuit mainly consists of the AFE for PPG and ECG signal acquisition, the MCU, and the Bluetooth transmission module. The AFE for the PPG signal is the MAX30102 due to its high SNR and ultra‐low power operation. Meanwhile, the AFE for the ECG circuit uses the AD8232, known for its low supply current (170 µA) and high immunity to interference, achieved through an internally integrated filter and amplifier. The AD8232 connects to three electrodes: the positive and negative electrodes are placed on the back of the patch for skin contact.

We selected the STM32F412ZGT6 for the MCU because of its storage capacity (1 MB Flash, 256 KB RAM), extensive peripheral options, and low power consumption. The MCU handles initial processing of the synchronously collected PPG and ECG signals, couples them into a PPG‐ECG signal, and transmits it to the terminal via Bluetooth Low Energy for display and monitoring. The hardware system is powered by a 3.3 V lithium battery.

### 4.2 Fabrication of a Chest Wearable Sensor Patch

Polyester fabric (XLCR2922TT, Coresport Textile Co., Ltd., Dongguan, China) was cut into specific patterns using laser‐cutting to serve as the substrate for the electrodes. The electrode traces and interfaces were printed onto the polyester substrate using the screen‐printing method (Figure S10). First, a custom‐designed screen mesh with specific patterns was placed on the polyester substrate, and conductive Ag/AgCl paste (CI‐1036, Engineered Materials Systems Inc.) was applied to the screen mesh. The paste was then spread evenly with a rubber squeegee. Finally, the Ag/AgCl electrode printed on the polyester substrate was placed in an oven at 65°C for 1 h to cure the Ag/AgCl paste.

A layer of thermoplastic polyurethane (TPU) film was applied onto the electrode traces as an insulating layer and was precisely laser‐cut to match the electrode trace pattern. It was then aligned with the Ag/AgCl substrate and heated at 120°C for 12 s to bond to the substrate. The electrode interface is covered with a commercial hydrogel patch (A001, Jun Lai Medical Co., Ltd., Dongguan, China), which lowers the impedance between the skin and the electrode and maintains signal quality.

The patch housing is designed to follow the device's contour, ensuring full exposure of electrode interfaces and the PPG sensors. The housing was fabricated via fused deposition modeling (FDM) using acrylonitrile butadiene styrene (ABS). The circuit and battery were aligned, inserted into the housing, and finally secured permanently with a pressure‐sensitive adhesive to ensure a robust assembly.

### 4.3 Design of the Signal Coupling Method

The specific method for coupling ECG and PPG signals is designed as follows. To preserve the phase information of the original signal after superposition and avoid the influence of QS waves in the ECG signal on the phase characteristics of the coupled signal, the MCU uses the dynamic threshold difference method (Note S4) to detect R waves in the ECG signal. Then, the points that do not meet the dynamic threshold condition are set to zero. These processed ECG and PPG signals are then combined into a single, coupled PPG–ECG signal via a weighted superposition as shown in Equation (1).

(1) PPG−EPG=α·PPG+β·ECG

The amplitude of the processed PPG signal is approximately twice that of the ECG signal. The weighting coefficients α and β are set to 0.5 and 1, respectively, to ensure both signals' characteristics are adequately represented in the coupled waveform (Figure S5). Subsequently, the high‐SNR R waves in the coupled signal serve as reliable temporal anchors for identifying PPG fiducial points. Within each RR interval, a constrained search window (from 0.1 to 0.9 times the RR interval) is applied to locate the local minimum, marking the PPG onset point. The systolic peak is then identified as the local maximum within the physiological window spanning from this onset point to the subsequent R wave. This R wave anchored approach significantly enhances the accuracy of fiducial point extraction and ensures precise temporal alignment of the two signals' time series.

### 4.4 Coupling of PPG‐ECG Signals for BP Estimation

The BP estimation model uses the PPG‐ECG coupling method, which involves several sequential steps. First, the patch circuit stores a segment of coupled PPG and ECG signals from the sensor patch. The PPG signal is aligned with the R wave of the ECG signal. To remove baseline drift and high‐frequency noise, the PPG‐ECG coupled signal is processed with a zero‐phase bandpass filter (0.5 – 40 Hz). During data processing, the MCU couples the PPG and ECG signals, and the coupled signals are transmitted to a host‐side buffer of the terminal. A sliding window calculates moving averages of waveform features, which are fed into the BP estimation model at 5 s intervals. The estimation of SBP and DBP uses different machine learning models.

### 4.5 Machine Learning Algorithms for BP Estimation

A total of 1574 data samples were collected from 24 healthy volunteers (13 males and 11 females) to develop the model, as detailed in Note S12, Tables S5 and S6. We used volunteer IDs to strictly separate the training and test sets, ensuring that all data from any individual volunteer belonged to only one set. The mean PTT was calculated for each volunteer. Based on their mean PTT values, volunteers were stratified into three tiers (short, medium, and long PTT). Within each tier, volunteers were allocated to the training set and test set in an 8:2 ratio. Ultimately, the training set comprised 80% of volunteers (*n* = 19), while the test set comprised 20% of volunteers (*n* = 5). The training set was then used to train separate models for SBP and DBP. To evaluate performance improvement from the signal coupling method, corresponding models were also trained using features derived solely from individual PPG and ECG signals, following the same procedure.

### 4.6 Application in Practical BP Estimation

We recruited another 10 healthy volunteers (5 males and 5 females) for practical BP estimation (Tables S5 and S7). During the dynamic BP assessment, volunteers wore the sensor patch on the chest and used an electronic sphygmomanometer (Omron 8102K) on the left upper arm (Figure S11). The BP measured by the sphygmomanometer was used as the ground truth BP. Volunteers rested for 5 min before the BP assessment. Each person was measured at least 20 times, with a minimum interval of 2 min between measurements. Volunteers were required to alter their BP through exercise. The above process was measured using coupled PPG‐ECG signals and independent PPG and ECG signals for comparison. Experiments involving human participants in this study were approved by the Medical Ethics Committee of Sichuan University, with approval number K2024014.

### 4.7 Statistical

The raw PPG‐ECG coupled signal was pre‐processed with a zero‐phase bandpass filter (0.5–40 Hz) to remove noise and drift. The interquartile range of BP for each volunteer was calculated, and outliers were excluded using Tukey's fence. Nonlinear transformation of the waveform features was applied, as detailed in Note S10.

All analyses were performed using Python 3.12. Data are primarily presented as mean ± standard deviation unless otherwise noted. The sample size (n) for each experiment represents the number of individual measurements as shown in the figure legends. Shapiro‐Wilk and Levene's tests were used to assess data normality and homogeneity of variance. The measured PTT and BP values from volunteers did not satisfy assumptions of normal distribution and homoscedasticity (*p* > 0.05). For PTT calculation accuracy across three methods, the non‐parametric Kruskal‐Wallis test was used to evaluate overall differences among the methods (*p* < 0.05). Mann‐Whitney U post‐hoc tests were then used to identify specific between‐group differences. For comparisons of BP estimation between the coupled PPG‐ECG signal and the individual signal, the Wilcoxon signed‐rank test was applied. All tests were two‐sided, and Bonferroni corrections were used to adjust for multiple comparisons. P values < 0.05 were considered statistically significant.

## Conflicts of Interest

The authors declare no conflicts of interest.

## Supporting information




**Supporting File**: advs74342‐sup‐0001‐SuppMat.docx.

**Supporting File**: advs74342‐sup‐0002‐SuppMat.pdf.

## Data Availability

The data that support the findings of this study are available from the corresponding author upon reasonable request.
