# Supplementary material for: Intrinsic PPG–ECG Coupling for Accurate and Low‐Power Blood Pressure Monitoring
Source: Adv Sci (Weinh). 2026 Mar 3;13(22):e20101. doi: 10.1002/advs.202520101 (PMC13088271; doi:10.1002/advs.202520101)
Supplement: Supplementary file 2 — Supporting File: advs74342‐sup‐0002‐SuppMat.pdf. [file ADVS-13-e20101-s002.pdf]

Supporting Information

Intrinsic PPG–ECG Coupling for Accurate and Low-Power Blood Pressure Monitoring

Sitong Chen[#], *Hua Luo*[#], *Zhentao Yao, Zhou Jiang, Xiaodong Wu*[*], *and Hanmin Liu*[*]

Sitong Chen
Department of Pediatric Pulmonology and Immunology, West China Second University Hospital, Sichuan University, Chengdu, China

Dr. Hua Luo, Zhentao Yao, Dr. Xiaodong Wu.
School of Mechanical Engineering, Sichuan University, Chengdu, China
E-mail: xiaodong_wu@scu.edu.cn (X. Wu)

Dr. Zhou Jiang
NHC Key Laboratory of Chronobiology, Sichuan University, Chengdu, China

Prof. Hanmin Liu

1. Department of Pediatric Pulmonology and Immunology, West China Second University Hospital, Sichuan University, Chengdu, China
2. Key Laboratory of Birth Defects and Related Diseases of Women and Children, Sichuan University, Ministry of Education, Chengdu, China
3. NHC Key Laboratory of Chronobiology, Sichuan University, Chengdu, China.
4. Department of Pediatric Pulmonology and Immunology, WCSUH-Tianfu·Sichuan Provincial Children's Hospital, Sichuan University, Meishan, China
5. The Joint Laboratory for Lung Development and Related Diseases of West China Second University Hospital, Sichuan University and School of Life Sciences of Fudan University, West China Institute of Women and Children's Health, West China Second University Hospital, Sichuan University, Chengdu, China
6. Sichuan Birth Defects Clinical Research Center, West China Second University Hospital, Sichuan University, Chengdu, China

E-mail: liuhm@scu.edu.cn (H. Liu)

# These authors contributed equally to this work.

* Corresponding author.

Note S1. PPG-ECG coupled signal pre-processing

The coupled PPG-ECG signals were processed after collection and storage at the MCU. The original PPG-ECG signal is first smoothed after acquisition using a 4-point moving average filter to reduce high-frequency noise. Next, a FIR bandpass filter based on a Hamming window is designed. The filter parameters are set with a pass-band frequency range of 0.5 to 40 Hz. A 0.5 Hz high-pass cutoff is used to remove baseline drift, while a 40 Hz low-pass cutoff reduces interference and noise from the working frequency range. The filter coefficients were computed using the standard windowing method for FIR design, with a Hamming window of order 8, a sampling frequency of 200 Hz, and the designated pass-band. The filter coefficients are converted into Q-point fixed-point format and stored directly in the microcontroller unit (MCU) to reduce computational overhead and improve real-time processing efficiency. The bandpass filtering is implemented via convolution of the PPG-ECG signal with these precomputed FIR coefficients, as expressed in **Equation (1)**.

$$y[n] = \sum_{k=0}^{N} h[k] * x[n-k] \quad (1)$$

where, h[k] represents the filter coefficient. x[n] is the *nth* sample of the input signal, and y[n] is the *nth* sample of the output signal.

Note S2. PPG and ECG are acquired simultaneously

To synchronize the acquisition of PPG and ECG signals, this study employs MAX30102's data-ready interruption as a hardware trigger. The MAX30102 is set to a sampling rate of 200 Hz and configured to generate a hardware interrupt signal upon each completion of sampling. This interrupt signal serves as an external trigger source for the internal AD converter of the MCU, which initiates the synchronous acquisition process of the ECG signal. Hardware triggering ensures that the PPG and ECG signals are temporally aligned during acquisition and have the same sampling frequency. This theoretically eliminates the temporal errors between the two signals.

Note S3. Signals pre-processing by analog front-end (AFE)

Because of the high signal-to-noise ratio and ultra-low power consumption of the MAX30102 (Maxim Integrated), it was chosen as the AFE for the photoelectric sensor. The photodiode (PD) converts changes in reflected light caused by blood volume into analog voltage signals, which the high-precision ADC then digitizes and stores in the MAX30102's data register. For the ECG AFE, the AD8232 was selected for its ultra-low static current (170 μA)

and excellent noise immunity. The integrated instrumentation amplifier and the driving right-leg circuit actively suppress common-mode interference, and the on-chip programmable filter limits the signal bandwidth to 0.5 - 40 Hz (**Figure S4**).

Note S4. Dynamic threshold difference method

1. Threshold initialization: Each ECG signal is segmented, and the data is divided into 4 equal segments. A differential operation is performed in each segment according to **Equation (2)**, with a differential step size of 1 selected. The differential minimum in each segment is detected, and the largest minimum is removed. The remaining differential minima are recorded as $\mathrm{C} = [\mathrm{C}_1, \mathrm{C}_2, \mathrm{C}_3]$, and the arithmetic average of the sequence C is taken as C_0. The initial threshold $\mathrm{Th1} = 0.5\mathrm{C}_0$. Using the fixed differential threshold $\mathrm{Th1}$, the positions of the R waves in each data segment are detected, and the RR intervals are calculated. The first three RR interval values are used to form the threshold sequence $\mathrm{RR} = [\mathrm{RR}_1, \mathrm{RR}_2, \mathrm{RR}_3]$, and the arithmetic average of the sequence RR is taken as RR_0.

$$\mathrm{y}' = \mathrm{ECG}(\mathrm{n} + \mathrm{t}) - \mathrm{ECG}(\mathrm{n}) \qquad (2)$$

2. R wave detection: If the differential signal $\mathrm{y}'_\mathrm{m} \leq \mathrm{Th1}$, then point *m* is identified as a potential R wave. Starting from position *m*, the local maximum within the interval [m - step, m + step] of the ECG signal is identified. The maximum point may correspond to an R wave position. The RR interval $\mathrm{RR}_{\mathrm{new}}$ is then calculated with the previous R wave. If the RR interval is determined to be $0.9\mathrm{RR}_0 \leq \mathrm{RR}_{\mathrm{new}} \leq 1.6\mathrm{RR}_0$. It is concluded that this point is an R wave.

3. When a new R wave is detected, the threshold update is initiated: the differential minimum of the detected signal in the interval [m, m+ step] is denoted as $\mathrm{C}_{\mathrm{new}}$, and a new RR interval $\mathrm{RR}_{\mathrm{new}}$ is computed. These values are used to update the threshold sequences for detecting the next cycle as C= $[\mathrm{C}_2, \mathrm{C}_3, \mathrm{C}_{\mathrm{new}}]$and,$\mathrm{rr} = [\mathrm{RR}_2, \mathrm{RR}_3, \mathrm{RR}_{\mathrm{new}}]$, respectively. The updated sequences C and rr are arithmetically averaged similarly to obtaining the new thresholds C_0 and RR_0, and the new threshold Th1=0.5*C_0 is calculated. To avoid noise interference, a forward delay of 200 ms is applied as a starting point after each R wave is detected, and the R waves for the next cycle are detected using the updated threshold according to step 2.[70-71]

Note S5. The establishment of the dedicated database

Synchronous PPG and ECG signals were recorded from 8 volunteers (4 males and 4 females, aged 18 to 33) in a seated, resting position. The ECG signals were acquired using a three-electrode configuration positioned on the chest to form a modified Lead I. The PPG

signals were obtained with a reflective-mode sensor firmly attached to the skin below the collarbone. Each recording included two channels: one PPG signal and one coupled PPG-ECG signal. The duration of the recordings ranged from 3 to 5 minutes. The PPG onset points and systolic peaks were annotated in each pulse signal using both the coupled and individual methods. We used the dynamic threshold difference method and manual confirmation to identify the R waves of the coupled signal, which ensures accurate anchor points.

Note S6. Local extremum extraction method of fiducial points

This method identifies characteristic points, such as systolic peaks and onset points, in PPG signals by locating local maxima and minima within physiologically constrained search windows. First, the original signal is bandpass-filtered with a 0.5–15 Hz FIR filter, and baseline drift is corrected using a moving average to produce high-quality signals for feature extraction. The core detection process starts by locating all local maxima, and candidate points are screened based on a feasible heart rate range (30–200 bpm) to identify all systolic peaks reliably, preliminarily. Then, using the position of the previously confirmed systolic peak as a reference, a dynamic search window is defined ahead of the current systolic peak. The most prominent local minimum designated as the corresponding onset point is found within this window.

Note S7. Adaptive derivative analysis to identify fiducial points

This method combines adaptive thresholding with first-derivative analysis to locate fiducial points in the PPG signal. First, the original signal is bandpass-filtered (0.5–15 Hz) using an FIR filter to minimize noise, and baseline drift is corrected using a moving average. The first derivative of the pre-processed signal is then calculated. For the diastolic segment of each cardiac cycle, a baseline mean (μ) and standard deviation (σ) are estimated within a quiescent interval. A dynamic threshold is set at $\mu + 1.5\sigma$. The pulse onset point is identified as the first point where the signal exceeds this threshold simultaneously with the derivative crossing zero from negative to positive. After the onset, the systolic peak is found at the following local maximum of the PPG signal, corresponding to where the derivative returns to zero after its positive peak.

Note S8. Fiducial points detection evaluation index

The detection accuracy of the fiducial points was assessed by conducting a point-by-point comparison against the manual reference annotations (gold standard) and the output of the PPG-ECG coupled signal method. From the detection results, we computed three quantitative

parameters: true positive (TP), when a fiducial point is correctly detected; false negative (FN), when a fiducial point is not detected; and false positive (FP), when a noise point is detected as a fiducial point. To assess the method's performance, the evaluation metrics include precision, recall, and F1 score, computed using **Equations (3)-(5)**.

$$\text{Precision} = \frac{\text{TP}}{\text{TP+FP}} \quad (3)$$

$$\text{Recall} = \frac{\text{TP}}{\text{TP+FN}} \quad (4)$$

$$\text{F1} = 2 \times \frac{\text{Precision}\times\text{Recall}}{\text{Precision+Recall}} \quad (5)$$

Note S9. Performance comparison of signal coupling methods in public databases

We validated the effectiveness and accuracy of the PPG–ECG coupled method for fiducial points detection using coupled PPG–ECG signals from the SensSmartTech database. The SensSmartTech database, a sub-database of PhysioBank, provides a unique multiparametric dataset systematically recorded during resting and post-activity relaxation periods[72-73]. It contains the simultaneously recorded 338 30-second ECG and PPG signals from 32 volunteers. Recorded PPG signals are resampled to a sampling rate of 500 Hz, the same as the ECG signals. Detection results for PPG systolic peaks and onset points from the SensSmartTech database are summarized in **Table S4**. Among all methods evaluated, the coupling method demonstrated superior performance for PTT estimation within this database.

Note S10. Feature selection for blood pressure estimation model

This study selected pulse transit time (PTT), pulse intensity ratio (PIR), rising time (T), and RR interval extracted from PPG–ECG coupled signals as key waveform features for the blood pressure (BP) estimation model. PTT indicates the physical properties of blood vessels, such as vascular wall thickness and blood density, which influence the pulse wave's propagation velocity. Because the stress state of the vascular wall changes instantly with BP, PTT is highly correlated with short-term BP fluctuations.[74-75] The PIR, calculated as the ratio of the peak amplitude to the trough amplitude of the PPG wave within one cardiac cycle, effectively reflects the regulatory effect of sympathetic nerve-mediated changes in arterial diameter on BP.[76-77] To further reduce the interference of cardiac activity on PTT and BP estimation, the RR interval and T were included in the model to improve its robustness.[75] To capture potential nonlinear relationships, we further expanded the feature set by including feature transformations (e.g., log(PTT), log(PIR), PTT^2, and PIR^2) and interaction terms between features (e.g., PTT*PIR, PIR/T). Finally, all these features were combined with physiological characteristics (weight,

age, and gender) to construct a multiple-feature model that provides more comprehensive and accurate BP estimates.[78-80]

Note S11. Performance metrics

The mean absolute error (MAE) was used to evaluate the accuracy of the estimated parameters, as shown in **Equation (6)**. The Cal_i is the calculation value, Ref_i, is the reference value, and n is the number of samples.

$$MAE = \frac{1}{n}\sum_{i=1}^{n} |Cal_i - Ref_i| \qquad (6)$$

The root mean square error (RMSE) is used to evaluate the accuracy of BP prediction model performance, as shown in **Equation (7)**.

$$RMSE = \sqrt{\frac{1}{n-1}\sum_{i=1}^{n}(y_i - x_i)^2} \qquad (7)$$

Where y_i is the prediction value, x_i is the reference value, and n is the number of samples.

Mean error (ME) is the average of the errors of all estimates and reference values. Let there be a total of n pairs of BP estimates BP_{est} and BP reference values BP_{ref} the mean error is defined as shown in **Equation (8)**.

$$ME = \frac{1}{n}\sum_{i=1}^{n}(BP_{ref,i} - BP_{est,i}) \qquad (8)$$

The standard deviation (STD) can be a measure of the dispersion of a set of data and is defined as shown in **Equation (9)**.

$$STD = \sqrt{\frac{1}{n}\sum_{i=1}^{n}(BP_{ref,i} - BP_{est,i} - ME)^2} \qquad (9)$$

The Pearson correlation coefficient is used to measure the linear correlation between two sets of data, and in this paper, it is calculated as shown in **Equation (10)**.

$$R = \frac{\sum_{i=1}^{n}(BP_{est,i} - \overline{BP}_{est})(BP_{ref,i} - \overline{BP}_{ref})}{\sqrt{(BP_{est,i} - \overline{BP}_{est})^2}\sqrt{(BP_{ref,i} - \overline{BP}_{ref})^2}} \qquad (10)$$

Note S12. Acquiring volunteer data for BP modelling

This study recruited 24 volunteers (13 males and 11 females) to develop the model. Volunteers remained rested for 5 minutes before the data acquisition. Physical characteristics (e.g., age, weight, gender) were recorded for all volunteers. Volunteers remained seated

throughout the measurement procedure. They wore the sensor patch on the chest and a cuff-based sphygmomanometer (Omron 8102K) on their left upper arm for simultaneous measurement (**Figure S11**). The BP measured by the cuff-based sphygmomanometer is used as a ground truth BP. Simultaneously, the sensor patch continuously recorded both the coupled PPG–ECG signals and individual PPG or ECG signals for subsequent comparison. We first measured volunteers' resting BP, then raised it with exercise, and monitored BP recovery to obtain a broader BP range. Each measurement procedure provides approximately 30 samples, with a minimum interval of 2 minutes between measurements. Each volunteer repeated the above measurement procedure once daily for two days. The BP were distributed as shown in **Figure S7**.

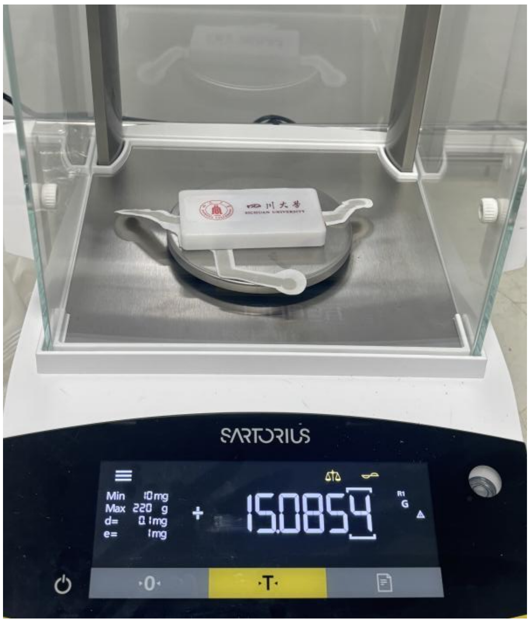

Figure S1. Picture of the chest wearable sensor patch. The entire chest wearable patch weighs only ≈15 g.

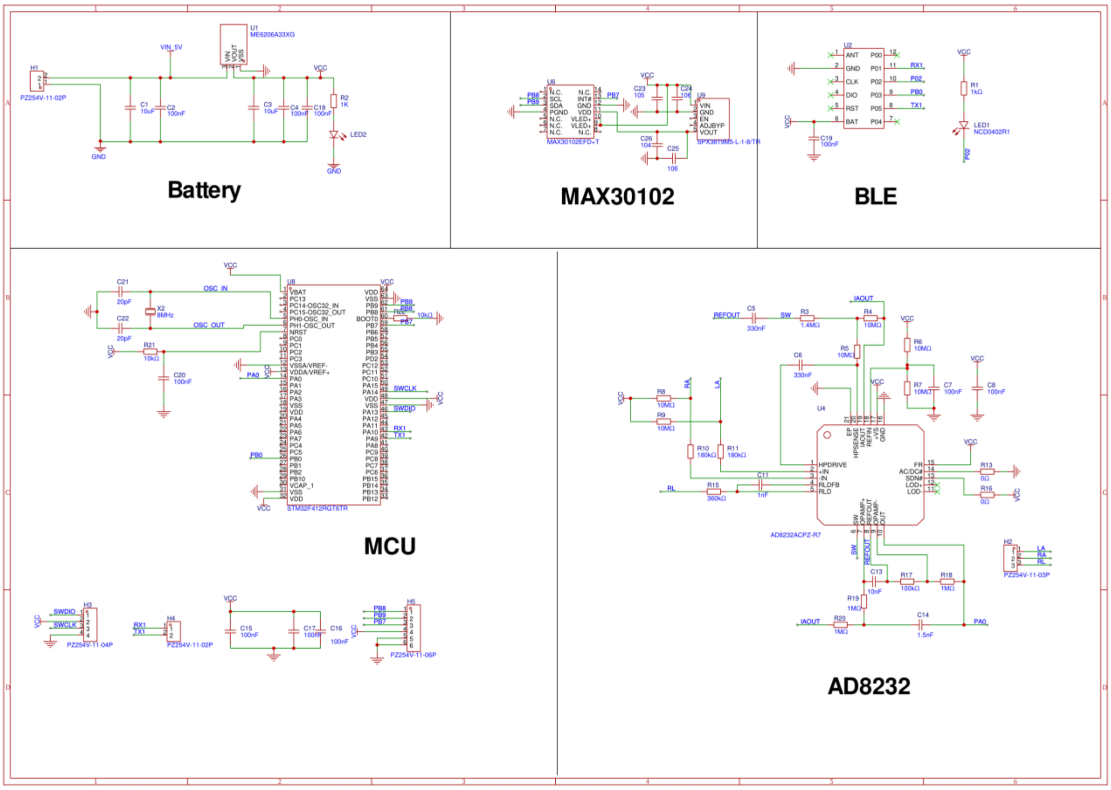

Figure S2. The schematic diagram of the sensor patch including the PPG and ECG signal acquisition modules, the MCU, a low-power Bluetooth module, and the power management unit.

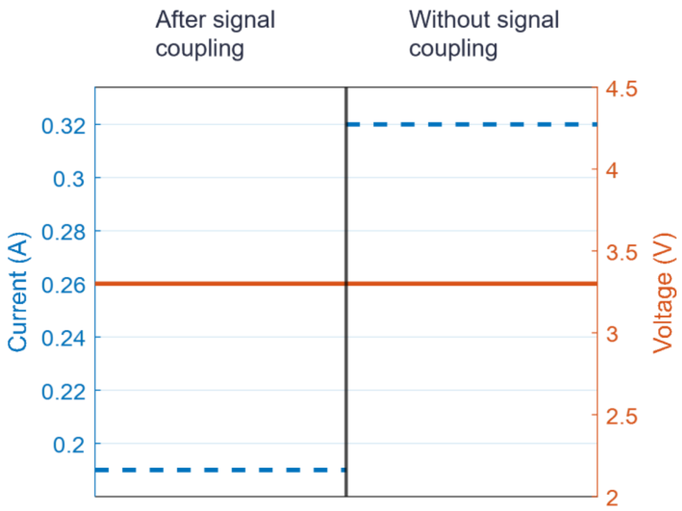

Figure S3. Power consumption comparison before and after signal coupling. Under a constant 3.3V supply voltage, the current consumption of the PPG-ECG coupled signal circuit was significantly reduced from 0.32A to 0.18A, resulting in a 43.75% reduction in power consumption, which is owed to the decreased data transmission requirements to the terminal.

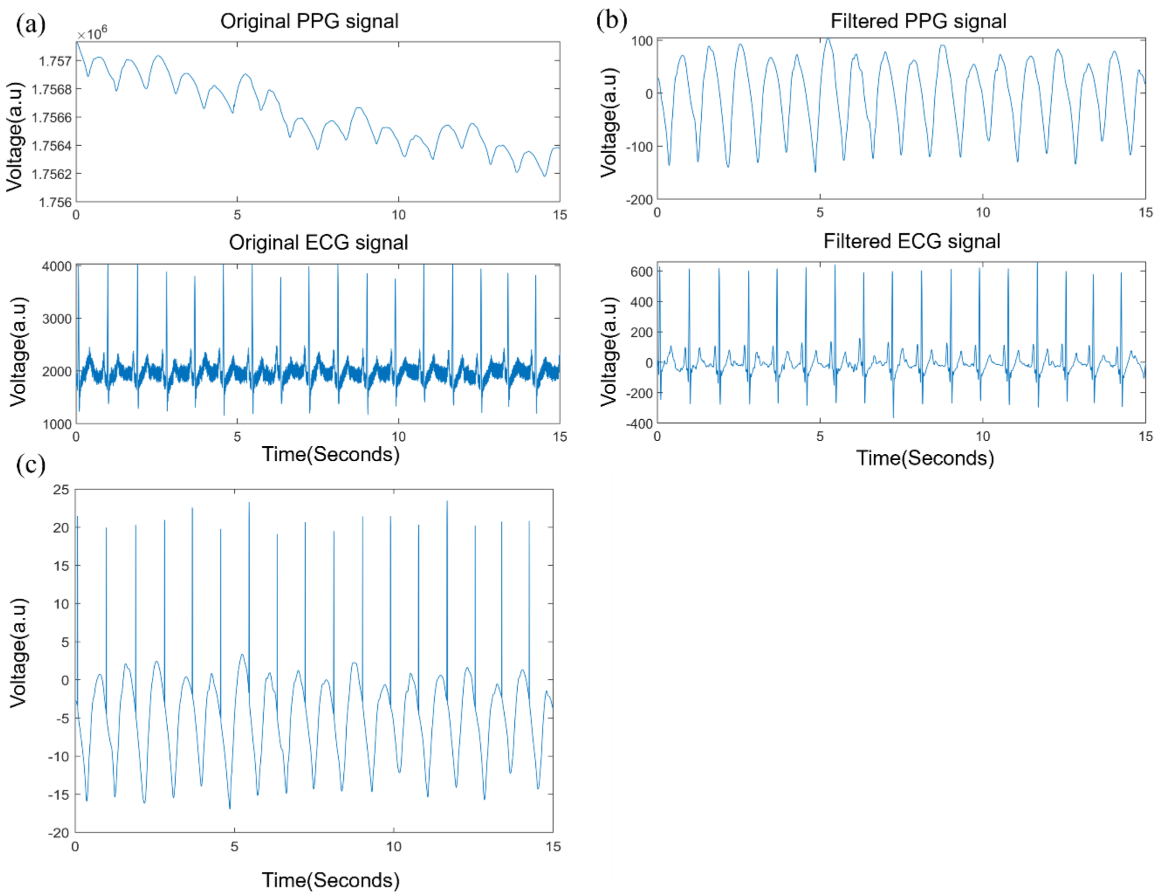

Figure S4. Signal pre-processing process. (a) Measured original PPG and ECG signals. (b) Filtered PPG and ECG signal. (c) PPG-ECG coupled signal. Processing the original signal with filtering and scaling yields the PPG-ECG coupled signal.

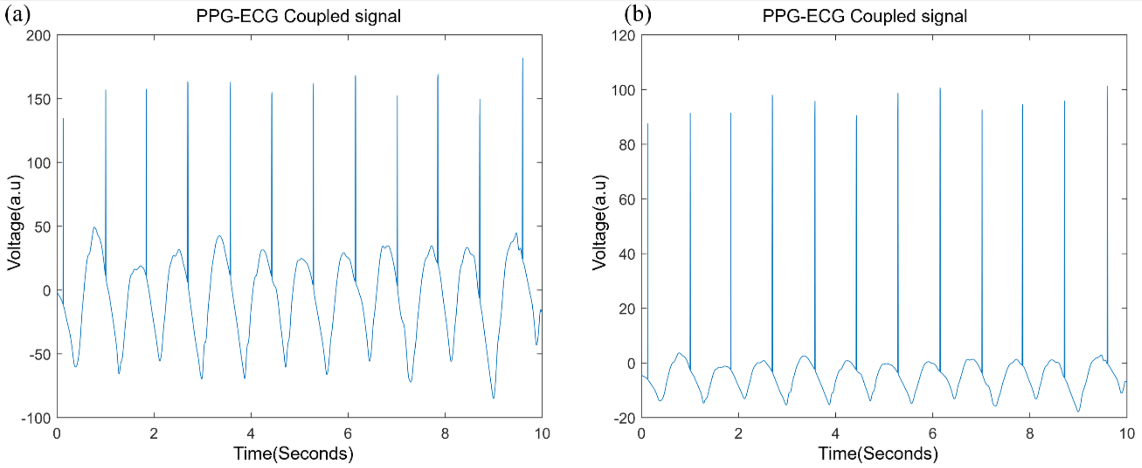

Figure S5. The PPG-ECG coupled signals under different weight coefficients based on $\mathrm{PPG-ECG} = \alpha \cdot \mathrm{PPG} + \beta \cdot \mathrm{ECG}$ (a) α=1 and β=0.5 (b) α=1 and β=2. When β=0.5, the relative weight of the PPG in the coupled signal increases, making its waveform features more prominent. Therefore, to enhance the discernibility of the PPG signal, the coefficient coupling with β=0.5 was selected.

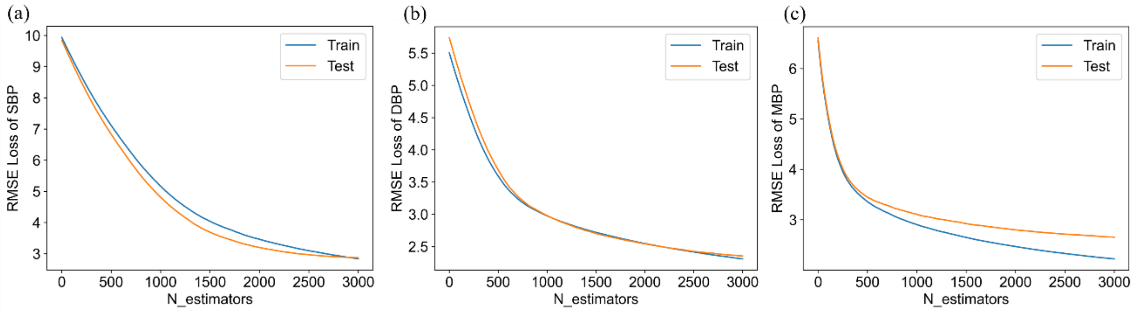

Figure S6. Loss curves during the model training. (a). Loss curves of DBP prediction as the estimator increases. (b). Loss curves of SBP prediction as the estimators increase. (c). Loss curves of MBP prediction as the estimator increases.

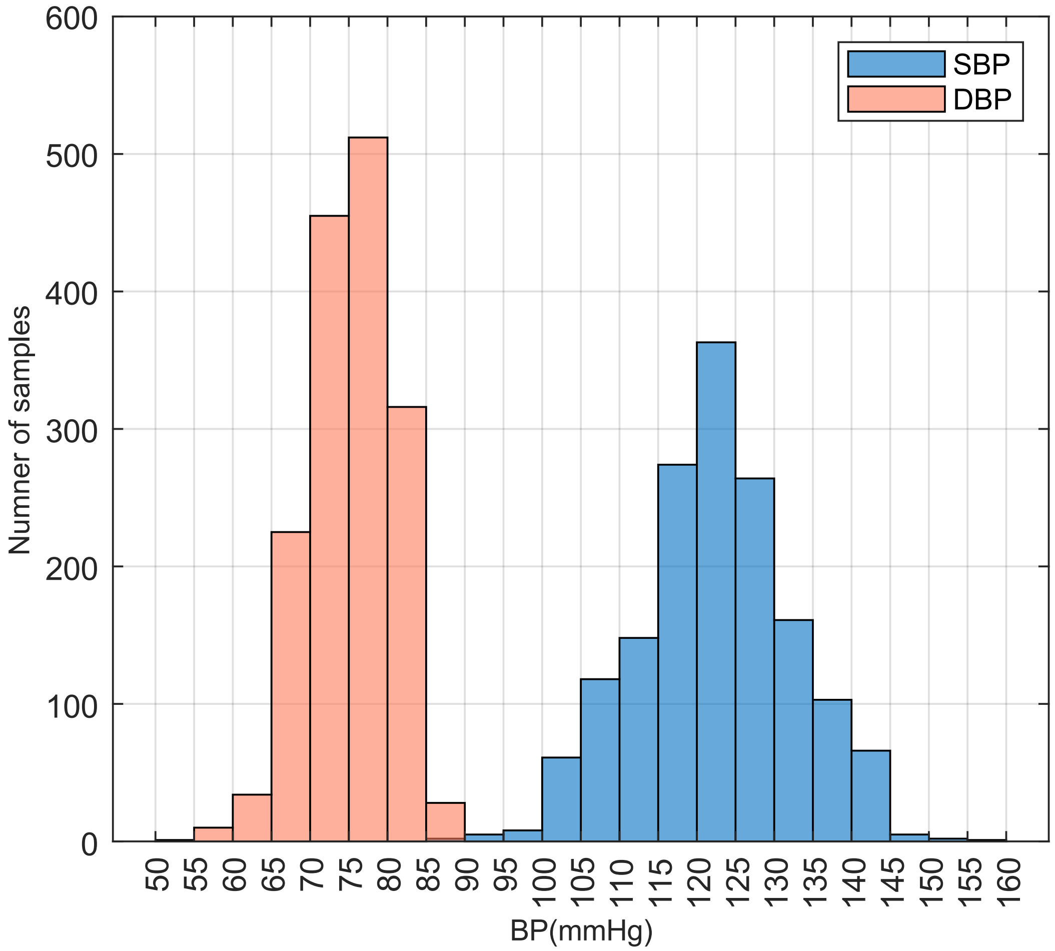

Figure S7. Distribution of BP. BP distribution across a wide range (SBP: 85–158 mmHg; DBP: 53–86 mmHg).

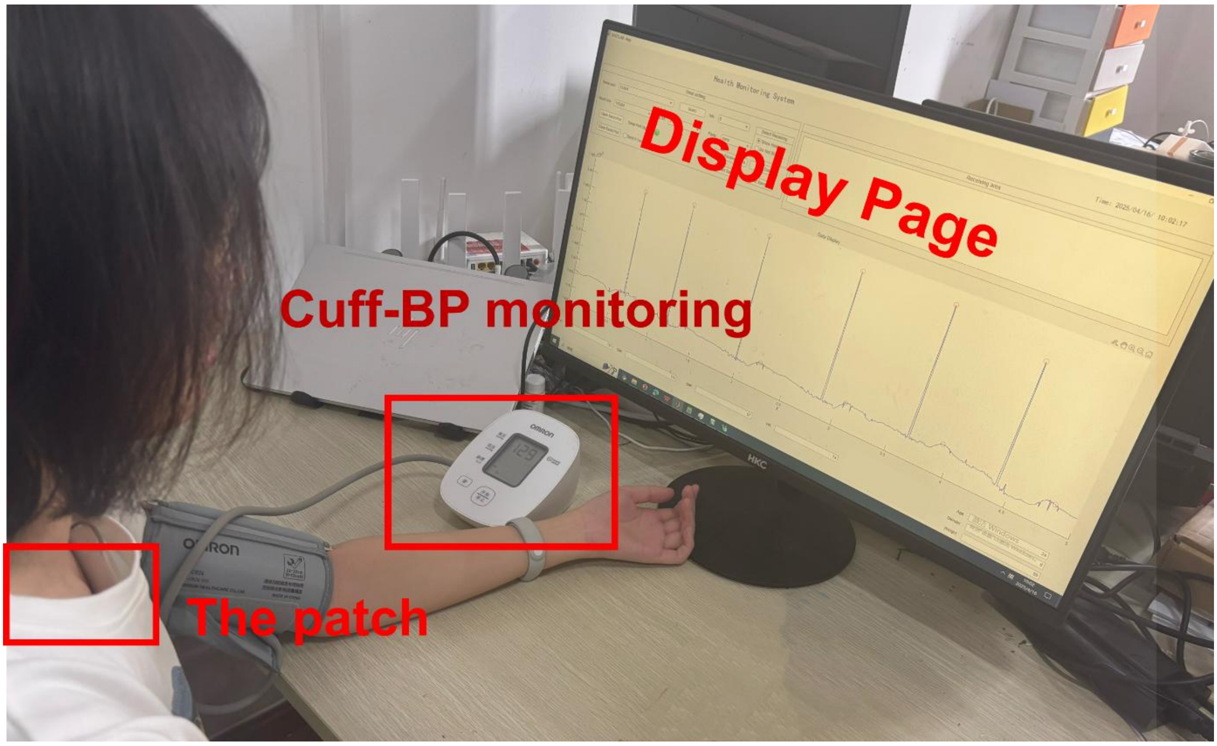

Figure S8. Photograph of BP measurement. BP was measured in volunteers using a sensor patch placed on the chest. At the same time, a cuff-based sphygmomanometer (Omron 8102K) was used on the left upper arm to provide reference values. The measurements from the sphygmomanometer were used as a benchmark to compare with the BP values from the chest wearable sensor patch.

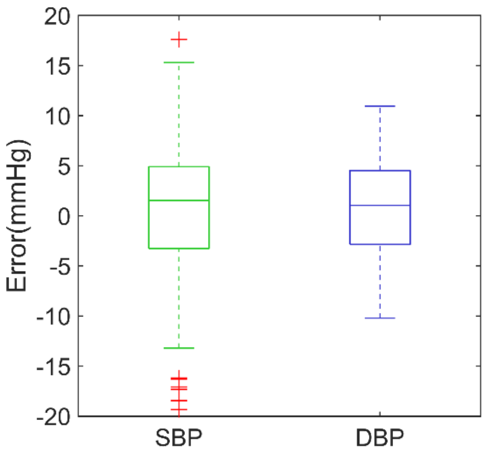

Figure S9. Box plot illustrates the estimation of BP error for the individual signal processing method. Median error for SBP is 1.544 mmHg, with an interquartile range (IQR) spanning from -3.255 mmHg to 4.906 mmHg. For DBP, the median error is 1.047 mmHg, with an IQR ranging from -2.847 mmHg to 4.519 mmHg.

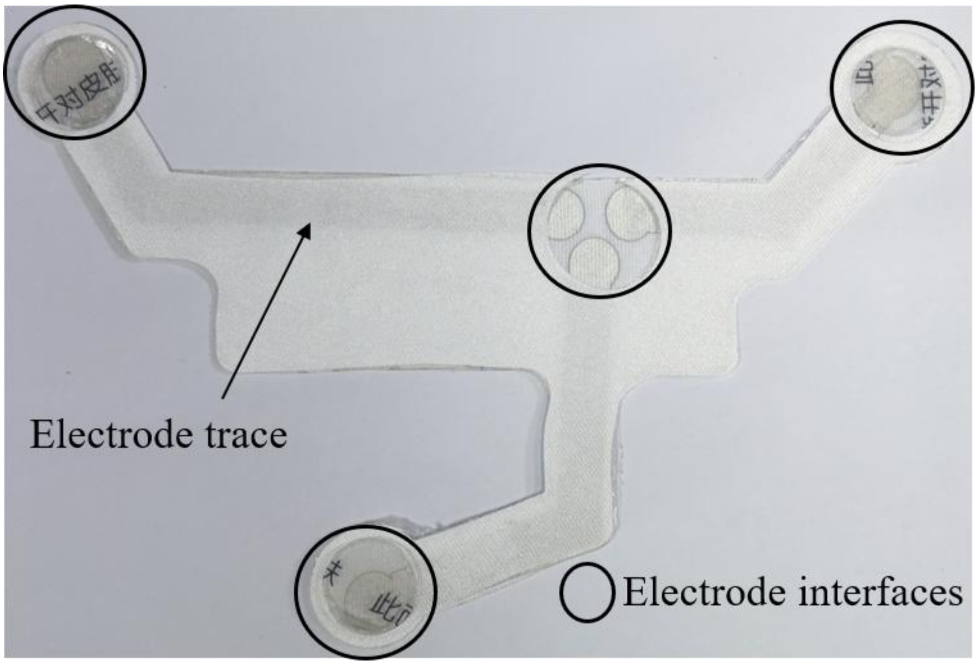

Figure S10. Printed electrodes for bio signal recording. The Ag/AgCl electrodes with the traces and interfaces were printed onto the polyester substrate. The circular regions correspond to the interfacial components, which are covered with hydrogel patches for skin contact and electrophysiological signal acquisition; the remaining areas form the conductive traces responsible for signal transmission. A TPU layer was applied over the traces for electrical isolation, followed by a polyester overlay for mechanical protection.

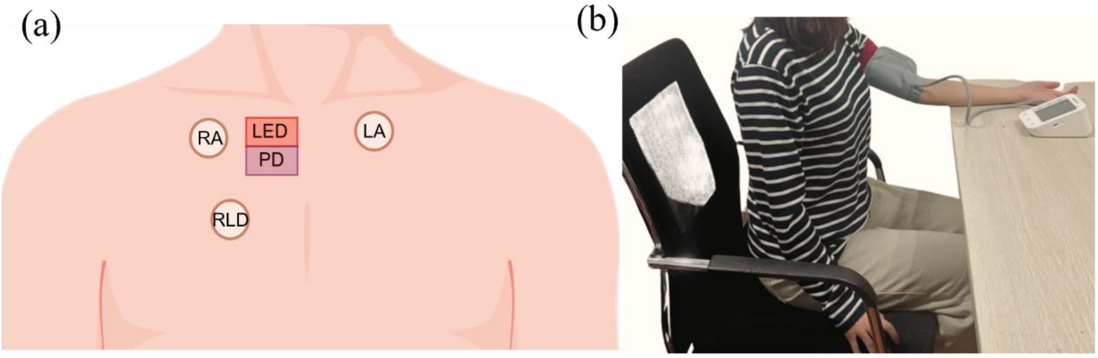

Figure S11. Details of the measurement. (a) Schematic of the sensor placement, showing the PPG sensor (LED and PD) positioned at the chest, surrounded by the three ECG electrodes (RA, LA, and RLD). (b) Illustration of the volunteer's seated and stationary posture maintained during the measurement.

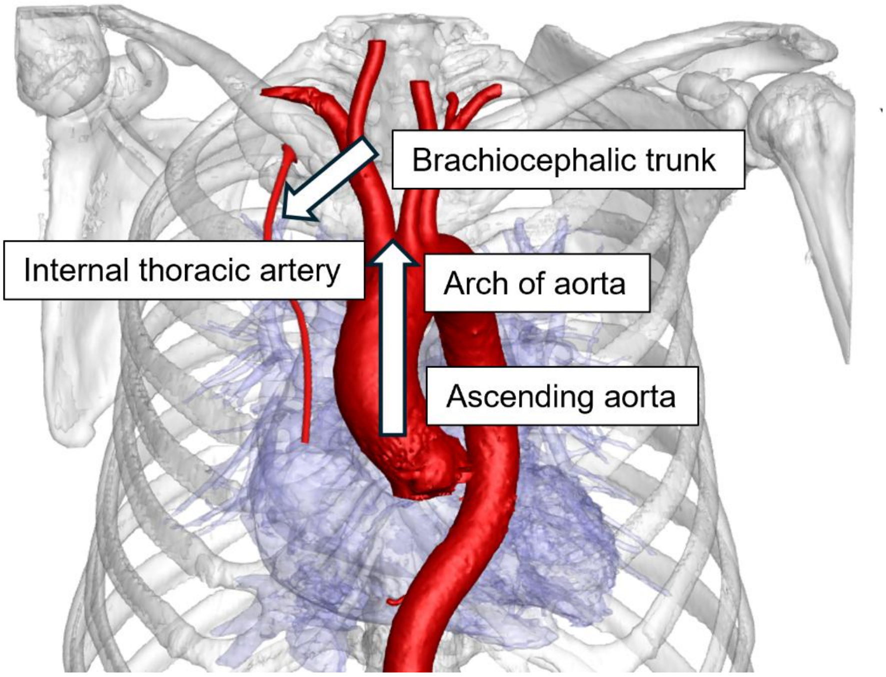

Figure S12. Anatomy of the arterial segments involved pulse wave propagation within the chest region. White arrows indicate the propagation direction of the pulses, including the large elastic arteries (ascending aorta, aortic arch, brachiocephalic trunk) and conductive arteries (internal thoracic artery).

Table S1. The state-of-the-art methods of wearable continuous BP measurement.

Method	Passive and continuous monitoring?	Signal transmission channel	Generalization	SBP estimation error	DBP estimation error	Ref
PWV	No. Relies on active user interaction (placing a finger on the watch)	Separate PPG and ECG signals	Model generalization across individuals[b)]	4.75±2.29mmHg (RMSE±STD)[a)]	2.72±0.75 mmHg (RMSE±STD)	[81]
PAT	Yes	Separate PPG and ECG signals	Subject-specific model[c)]	6.54mmHg (RMSE)	5.06mmHg (RMSE)	[82]
PWV	Yes	Multiple PPG signals	Subject-specific model	1.99±1.15mmHg (MAE±STD)	1.63±0.84mm Hg (MAE±STD)	[83]
PTT	No Unintegrated electronic sampling control equipment	3-channel of the Bio-Z Sensor Array	Model generalization across individuals	0.5±5.0mmHg (ME±STD)	0.2±6.5mmHg (ME±STD)	[84]
Piezoelectric dynamics	Yes. Sensors on fingers and arms connect to the wrist signal acquisition	2-channel piezoelectric responses	Not stated	Not stated		[85]
Ultrasound	Yes. Ultrasonic-system-on-patch	32-channel piezoelectric transducer array	Model generalization across individuals	95% of BP errors lie within -8.24 to 8.59 mmHg.		[86]
PTT	Yes	Separate PPG and ECG signals for training; Only PPG for application.	Subject-specific model	3.58±5.96mmHg (MAE±STD)	2.91±4.60mmHg (MAE±STD)	[87]
Physiological signal fusion map	No Non-real-time data processing.	Separate PPG and ECG signals	Subject-specific model	3.87mmHg (MAE)	3.59mmHg (MAE)	[88]
**PTT**	**Yes. Integrated patch measures ECG and PPG on the chest**	**One coupled PPG-ECG signal**	**Model generalization across individuals**	**4.08±5.46mmHg (MAE±STD)**	**2.69±3.19mm Hg (MAE±STD)**	**This work***

a)ME is the mean error. STD is the standard deviation. MAE is the mean absolute error. RMSE is standard root mean squared error; b) Model generalization across individuals refers to the generalized model that is trained and tested across different subjects; c)Subject-specific model refers to the individual model that is trained and tested by the same subject.

Table S2. Performance comparison of different methods for fiducial point identification in dedicated dataset.

	Systolic peak			Onset point		
	**Prec**	**Rec**	**F1 Score**	**Prec**	**Rec**	**F1 Score**
PPG-ECG Coupling	0.959	0.966	0.962	0.982	0.971	0.977
Local Extrema Detection	0.769	0.782	0.776	0.943	0.940	0.942
Derivative Analysis	0.715	0.713	0.714	0.726	0.959	0.826

Table S3. Fiducial point identification performance of PPG-ECG coupled method on the dedicated dataset.

	Systolic Peak						Onset Point					
**Record**	**TP**	**FP**	**FN**	**Pre**	**Rec**	**F1**	**TP**	**FP**	**FN**	**Pre**	**Rec**	**F1**
Record01	287	10	14	0.966	0.953	0.960	300	9	10	0.971	0.968	0.969
Record02	382	15	9	0.962	0.977	0.970	395	6	8	0.985	0.980	0.983
Record03	229	14	8	0.942	0.966	0.954	235	8	5	0.967	0.979	0.973
Record04	319	13	11	0.961	0.967	0.964	312	8	15	0.975	0.954	0.964
Record05	309	9	12	0.972	0.963	0.967	320	6	14	0.982	0.958	0.970
Record06	367	10	14	0.973	0.963	0.968	364	5	13	0.986	0.966	0.976
Record07	252	13	8	0.951	0.969	0.960	265	3	3	0.989	0.989	0.989
Record08	275	19	10	0.935	0.965	0.950	300	0	6	1.000	0.980	0.990
**Overall**	**2420**	**103**	**86**	**0.959**	**0.966**	**0.962**	**2491**	**45**	**74**	**0.982**	**0.971**	**0.977**

Table S4. Fiducial points identification performance in the public database (SensSmartTech).

	Systolic peaks			Onset points		
	Prec	Rec	F1 Score	Prec	Rec	F1 Score
PPG-ECG Coupling	0.972	0.957	0.965	0.969	0.961	0.965
Local Extrema Detection	0.921	0.815	0.865	0.725	0.957	0.824
Derivative Analysis	0.944	0.619	0.747	0.841	0.860	0.850

Table S5. Summary of volunteer characteristics and participation in experiments.

No.	Weight (kg)	Age	Gender (Female/Male)	Model development		The sensor validation experienements		Feature Identification
				Train	Test	Ambulatory BP monitoring	Long-term BP test	
1	54	26	Female	√				
2	52	31	Female	√				
3	67	33	Male	√				√
4	74	27	Male	√				
5	68	20	Male	√				
6	66	26	Male	√				
7	72	51	Male		√			
8	64	53	Male		√			
9	67	28	Male	√				
10	68	21	Male	√				√
11	89	24	Male		√			√
12	66	25	Female	√				√
13	78	28	Male	√				
14	65	19	Female	√				
15	72	20	Male	√				
16	57	29	Female		√			
17	59	38	Female	√				
18	54	27	Female	√				
19	67	29	Male	√				
20	52	25	Female	√				√
21	49	55	Female		√			
22	70	28	Female	√				
23	71	23	Male	√				
24	56	22	Female	√				
25	47	20	Female			√	√	
26	82	17	Male			√	√	
27	90	18	Male			√	√	√
28	67	21	Male			√	√	
29	57	22	Female			√	√	√
30	56	24	Female			√	√	
31	66	26	Male			√	√	
32	58	28	Female			√	√	√
33	73	31	Male			√	√	
34	56	33	Female			√	√	

Table S6. Demographic characteristics of volunteers for model training and evaluation. They vary in gender, age, and weight, which generates diversity in the collected BP information for model training.

Gender	N (percentage)
Male	13 (53.3%)
Female	11 (46.6%)
**At the time of study**	**Mean ± Standard deviation**
Age (years)	29.50 ± 9.43
Weight (kg)	64.75 ± 10.06

Table S7. Demographic characteristics of volunteers for ambulatory BP monitoring and long-term stability validation.

Gender	N (percentage)
**Male**	5 (50%)
**Female**	5 (50%)
**At the time of study**	**Mean ± Standard deviation**
**Age (years)**	23.8 ± 5.47
**Weight (kg)**	65.2 ± 13.26

Reference

[70] X. Gu, J. Hu, L. Zhang, J. Ding, F. Yan. An Improved Method with High Anti-interference Ability for R Peak Detection in Wearable Devices. IRBM, 2020, 41(3): 172. https://doi.org/https://doi.org/10.1016/j.irbm.2020.01.002

[71] H. Dogan, R. O. Dogan. A Comprehensive Review of Computer-based Techniques for R-Peaks/QRS Complex Detection in ECG Signal. Archives of Computational Methods in Engineering, 2023, 30(6): 3703. https://doi.org/10.1007/s11831-023-09916-x

[72] A. L. Goldberger, L. A. N. Amaral, L. Glass, J. M. Hausdorff, P. C. Ivanov, R. G. Mark, et al. PhysioBank, PhysioToolkit, and PhysioNet. Circulation, 2000, 101(23): e215. https://doi.org/10.1161/01.CIR.101.23.e215

[73] Lazović, A., Tadić, P., Đorđević, N., Atanasoski, V., Tiosavljevic, M., Ivanovic, M., Hadzievski, L., Ristic, A., Vukcevic, V., and Petrovic, J. (2024) 'SensSmartTech database of cardiovascular signals synchronously recorded by an electrocardiograph, phonocardiograph, photoplethysmograph and accelerometer' (version 1.0.0), PhysioNet. RRID:SCR_007345. https://doi.org/10.13026/fy9p-n277

[74] H. Gesche, D. Grosskurth, G. Küchler, A. Patzak. Continuous blood pressure measurement by using the pulse transit time: comparison to a cuff-based method. European journal of applied physiology, 2012, 112(1): 309. https://doi.org/10.1007/s00421-011-1983-3

[75] A. Bringard, A. Adami, N. Fagoni, T. Fontolliet, F. Lador, C. Moia, et al. Dynamics of the RR-interval versus blood pressure relationship at exercise onset in humans. European journal of applied physiology, 2017, 117(4): 619. https://doi.org/10.1007/s00421-017-3564-6

[76] X.-R. Ding, Y.-T. Zhang, J. Liu, W.-X. Dai, H. K. Tsang. Continuous cuffless blood pressure estimation using pulse transit time and photoplethysmogram intensity ratio. IEEE Transactions on Biomedical Engineering, 2015, 63(5): 964. https://doi.org/10.1109/TBME.2015.2480679

[77] C. Fischer, T. Penzel. Continuous non-invasive determination of nocturnal blood pressure variation using photoplethysmographic pulse wave signals: comparison of pulse propagation time, pulse transit time and RR-interval. Physiological measurement, 2019, 40(1): 014001. https://doi.org/10.1088/1361-6579/aaf298

[78] M. Gao, N. B. Olivier, R. Mukkamala. Comparison of non-invasive pulse transit time estimates as markers of blood pressure using invasive pulse transit time measurements as a reference. Physiological reports, 2016, 4(10). https://doi.org/10.14814/phy2.12768

[79] A. Cisnal, Y. Li, B. Fuchs, M. Ejtehadi, R. Riener, D. Paez-Granados. Robust Feature Selection for BP Estimation in Multiple Populations: Towards Cuffless Ambulatory BP Monitoring. IEEE journal of biomedical and health informatics, 2024, 28(10): 5768. https://doi.org/10.1109/jbhi.2024.3411693

[80] S. Tian, L. Wang, R. Zhu. A flexible multi-modal pulse sensor for wearable continuous blood pressure monitoring. Materials horizons, 2024, 11(10): 2428. https://doi.org/10.1039/d3mh01999c

[81] V. G. Ganti, A. M. Carek, B. N. Nevius, J. A. Heller, M. Etemadi, O. T. Inan. Wearable cuff-less blood pressure estimation at home via pulse transit time. IEEE journal of biomedical and health informatics, 2020, 25(6): 1926. https://doi.org/10.1109/JBHI.2020.3021532

[82] T. Y. Wu, F. Wu, C. K. Qiu, J. M. Redoute, M. R. Yuce. A Rigid-Flex Wearable Health Monitoring Sensor Patch for IoT-Connected Healthcare Applications. Ieee Internet of Things Journal, 2020, 7(8): 6932. https://doi.org/10.1109/jiot.2020.2977164

[83] Z. C. Liu, C. Xiang, Y. Y. Tong, K. H. Li, X. Guan. Transfer Learning Enhanced Blood Pressure Monitoring Based on Flexible Optical Pulse Sensing Patch. Acs Sensors, 2025, 10(4): 2732. https://doi.org/10.1021/acssensors.4c03404

[84] B. Ibrahim, R. Jafari. Cuffless blood pressure monitoring from a wristband with calibration-free algorithms for sensing location based on bio-impedance sensor array and autoencoder. Scientific Reports, 2022, 12(1). https://doi.org/10.1038/s41598-021-03612-1

[85] Z. R. Yi, Z. X. Liu, W. B. Li, T. Ruan, X. Chen, J. Q. Liu, et al. Piezoelectric Dynamics of Arterial Pulse for Wearable Continuous Blood Pressure Monitoring. Advanced Materials, 2022, 34(16). https://doi.org/10.1002/adma.202110291

[86] M. Y. Lin, Z. Y. Zhang, X. X. Gao, Y. Z. Bian, R. S. Wu, G. Park, et al. A fully integrated wearable ultrasound system to monitor deep tissues in moving subjects. Nature Biotechnology, 2024, 42(3). https://doi.org/10.1038/s41587-023-01800-0

[87] N. Murmu, R. Gupta, K. D. Sharma. Real-Time PPG-to-ECG Reconstruction Model With On-Device Recalibration Facility. IEEE Transactions on Instrumentation and Measurement, 2024, 73: 1. https://doi.org/10.1109/TIM.2024.3450120

[88] H. Wang, M. Han, C. Zhong, C. Wang, R. Chen, G. Zhang, et al. Non-invasive continuous blood pressure prediction based on ECG and PPG fusion map. Medical Engineering & Physics, 2023, 119: 104037. https://doi.org/https://doi.org/10.1016/j.medengphy.2023.104037
